# Complications, compliance, and undertreatment do not explain the relationship between cognition and survival in diffuse glioma patients

**DOI:** 10.1093/nop/npac027

**Published:** 2022-04-05

**Authors:** Emma van Kessel, Eva A Krijnen, Suzanne IJpelaar, Irene M C Huenges Wajer, Carla Ruis, Tatjana Seute, Filip Y F L De Vos, Joost J C Verhoeff, Pierre A Robe, Martine J E van Zandvoort, Tom J Snijders

**Affiliations:** Department of Neurology and Neurosurgery, University Medical Center Utrecht/UMC Utrecht Brain Center, Utrecht, the Netherlands; Department of Neurology and Neurosurgery, University Medical Center Utrecht/UMC Utrecht Brain Center, Utrecht, the Netherlands; Department of Neurology and Neurosurgery, University Medical Center Utrecht/UMC Utrecht Brain Center, Utrecht, the Netherlands; Experimental Psychology, Helmholtz Institute, Utrecht University, Utrecht, the Netherlands; Experimental Psychology, Helmholtz Institute, Utrecht University, Utrecht, the Netherlands; Department of Neurology and Neurosurgery, University Medical Center Utrecht/UMC Utrecht Brain Center, Utrecht, the Netherlands; Department of Medical Oncology, University Medical Center Utrecht/UMC Utrecht Brain Center, Utrecht,the Netherlands; Department of Radiation Oncology, University Medical Center Utrecht/UMC Utrecht Brain Center, Utrecht, the Netherlands; Department of Neurology and Neurosurgery, University Medical Center Utrecht/UMC Utrecht Brain Center, Utrecht, the Netherlands; Department of Neurology and Neurosurgery, University Medical Center Utrecht/UMC Utrecht Brain Center, Utrecht, the Netherlands; Department of Neurology and Neurosurgery, University Medical Center Utrecht/UMC Utrecht Brain Center, Utrecht, the Netherlands

**Keywords:** cognition, diffuse glioma, mediation-analyses, survival

## Abstract

**Background:**

Cognitive deficits occur in all different grades of glioma. In a recent study, we found these deficits to be independently, and possibly causally, related to survival in diffuse gliomas. In this study, we investigated whether the relationship between cognition and survival was mediated by three different factors: undertreatment, complications of treatment, and compliance. We hypothesized that patients with cognitive impairment may undergo less intensive treatment, be less compliant, and suffer more from complications, resulting in shortened survival for cognitively impaired patients.

**Methods:**

In a retrospective cohort study of patients undergoing awake craniotomy between operative neuropsychological assessments in five cognitive domains. We used Structural Equation Modeling to perform mediation analyses. Mediation analyses are analyses to evaluate whether a variable is a factor *in* the causal chain, referred to as an intermediate factor.

**Results:**

In total 254 patients were included, of whom 111 patients were LGG patients and 143 were HGG patients. The most frequently impaired domain was memory (37.8% *≤–2 SD*) in HGG and attention and executive functioning in LGG (33.3*≤–1.5 SD).* We confirmed the significant association between different cognitive domains and survival. These associations could not be explained by one of the aforementioned intermediate factors.

**Conclusions:**

This suggests that other mechanisms should be involved in the relation between cognition and survival. Hypothetically, cognitive functioning can act as a marker for diffuse infiltration of the tumor or cognitive functioning and survival could be determined by overlapping germline and somatic tumoral molecular-genetic factors.

Diffuse glioma (WHO grade II-IV) are progressive and invariably fatal brain tumors, which shorten life expectancy and compromise patients’ quality of life through neurological symptoms. A common and debilitating symptom is cognitive impairment, which is the result of multiple factors. Cognitive deficits occur in all different grades of glioma and are most prevalent in the domains executive functioning and memory.^1,2^ In a recent study, we found these deficits to be independently, and possibly causally, related to survival in diffuse gliomas.^3^ Possible mechanisms underlying this relationship include both biologically and clinically based factors. The notion of cognitive functioning as marker for diffuse tumor infiltration, and the option that cognitive functioning and survival are determined by overlapping genetic pathways and biomarkers might play a role as biological factors. Clinically based factors influencing patients’ cognitive impairments are therapeutic decision making, treatment compliance, and the risk of complications. In this way “undertreatment”, “compliance”, and “complications” would act as *intermediate factors* in the relationship between cognition and survival.

Although cognition is not included as formal criterion for postoperative treatment decision-making in most clinical guidelines, cognition could influence the choice of therapy. Possibly, physicians consider patients with severe cognitive problems to be less eligible for more intensive therapies. As such, the unfavorable prognosis of cognitively impaired patients may constitute a self-fulfilling prophecy, since physicians may base their treatment strategy on this published prognostic value of cognitive status (even though cognition is not necessarily predictive of treatment effect).

Regarding the other possible intermediate factors, cognitive impairment has been shown to be a predictor for poor compliance, possibly due to behavioral problems and/or difficulty in comprehending instructions of their medical team.^4,5^ As such, adherence to medication is negatively influenced by cognitive deficits. Behavioral problems and decreased comprehension may also increase the risk of complications. For example, patients with dementia have an increased risk of adverse healthcare events including increased morbidity and mortality, independently of age, sex, and disease duration.^6^ Poor compliance and complications, in turn, could shorten life expectancy.

To our knowledge therapeutic decision-making, compliance and complications really mediating the relationship between cognitive functioning and survival has not been investigated yet. In this retrospective cohort study, we aimed to investigate whether therapeutic decision-making, compliance, and complications act as intermediate factors in the established relationship between cognitive functioning and survival in treatment-naive patients with diffuse gliomas. We hypothesize that patients with cognitive impairment may undergo less intensive treatment, be less compliant, and suffer more from complications, resulting in shortened survival for cognitively impaired patients.

## Materials and Methods

### Design and Participants

We performed a single-center retrospective study in a cohort of treatment-naive diffuse glioma patients who underwent elaborate neuropsychological testing as part of their preoperative work-up for awake brain surgery between January 2010 and July 2019 at the University Medical Center Utrecht, The Netherlands (UMCU). We made use of an existing cohort as described in an earlier study^3^ and extended this cohort with 57 patients operated on between 2017 and 2019.

Inclusion criteria were the presence of a diffuse glioma, WHO grade II-IV, according to WHO 2016 criteria, and a minimum age of eighteen years old. For tumors diagnosed before 2016, we used all available histological and molecular data from clinical practice to (re-)classify the tumor according to WHO 2016 criteria. Exclusion criteria were

(a) any form of tumor-directed treatment—such as cytoreductive surgery, chemotherapy, radiotherapy—before neuropsychological assessment. Having undergone biopsy shortly before a planned resection was allowed.
(b) incomplete neuropsychological assessment (e.g. due to emergency surgery).

The UMCU institutional ethical review board approved the study; informed consent was not obtained for this observational study on data that were obtained as part of routine clinical care (protocol code METC 17/384 and 09-420)

### Neuropsychological Tests

In the study sample, we focused on neurocognitive functioning (NCF) scores for five predefined cognitive domains: executive functioning, memory, psychomotor speed, language, and visuospatial functioning. Neuropsychological instruments that were used as part of our routine clinical care are listed in Supplementary Table 1. These tests are internationally widely used, standardized psychometric instruments for assessing neurocognitive deficits.^7^ Details regarding assessment and evaluation of neuropsychological tests can be found in the “Methods Supplement”. Briefly, neuropsychological evaluation was conducted with the use of predetermined test classifications (Supplementary Table 1)^8–10^ shortly before surgery. Each neuropsychological test was scored according to standardized scoring criteria. For normative comparisons the unadjusted scores were transformed into Z-scores based on the mean and standard deviation of control subjects derived from published, age- and education-specific, norm data. For detailed description regarding NCF of our study population we refer to previously published data.^11^ In this study we decided to group tasks on their conceptual background in order to enhance power, as analyses per task would add up to an undesirable number of analyses. This choice inevitably leads to differences in number of tasks per cognitive domain. The question what cognitive concept (or domain) is best represented by a specific task is always complicated since intrinsically more than one concept is tapped in any task. However, neuropsychologists do share common ground in the categorization of tasks across domains. With respect to semantic fluency, we are convinced this is more associated with semantic memory than it is with either language or executive functioning.^9^ The findings of Biesbroek et al. indicate that anatomical correlates of semantic and phonemic fluency overlap in the left inferior frontal gyrus and insula (reflecting shared underlying cognitive processes as executive functioning and attention), however semantic fluency additionally draws on left medial temporal regions, probably reflecting a search through semantic memory.

For high-grade glioma (HGG), patients were considered impaired if they performed below –2 SD on any of the administered (sub)tests within a given domain.^12^ For low-grade glioma (LGG) patients, we used a threshold of –1.5 SD for cognitive deficits.^1,2^

### Data Collection—Baseline Data

All neuropsychological data were prospectively collected between 2010 and 2019. We further extracted data on patient characteristics from electronic patient files for all diffuse glioma patients undergoing awake surgery in this period. Data included sex, age at surgical resection, survival time and status, integrated (“layered”) histomolecular diagnosis based on WHO 2016 classification, MGMT-methylation status of the tumor, KPS, preoperative tumor volume, and neurologic deficits or epileptic seizures at presentation.^13,14^ Volumes were measured in 3D with use of Osirix Lite (v. 9.5.2) on T2-/fluid-attenuated inversion recovery (FLAIR)-weighted MRI scans. Volumes were defined as the whole area of hyperintensity. This represents the total lesion volume, including tumor infiltration and edema. Volumes were measured by a junior clinical scientist and neuro-oncological neurosurgeon with experience in preoperative tumor delineation. Since this parameter is independent of enhancement (and thereby grade) of the lesion, it forms a widely usable representation of the extent of brain volume that is potentially hampered in its function by the tumor in any way.^15^

### Data Collection—Follow-up Data

Follow-up data were extracted from electronic patient files, including data regarding treatment, compliance, complications, and survival. Survival time was defined as the period between first neurosurgery and the date of death from cancer or any other cause, or censored at the date of last follow-up (February 5, 2021).

To evaluate “undertreatment”, differences between treatment based on the guideline and treatment that was actually initiated based on the tumor board’s recommendation and shared decision-making process with the patient (referred to as “initiated treatment”), were reported as to whether patients received treatment in accordance with the guideline or received a decreased treatment intensity compared to the guideline. Follow-up treatment of each patient that should be given based on the guideline was determined by two authors (EAK, SIJ) who were blinded for the actually initiated treatment. They used flowcharts compiled from national guidelines^16^ and local specifications of these guidelines from the UMCU. In these flowcharts and guidelines, optimal treatment was based on the following criteria: WHO2016 glioma classification, age, KPS, Pignatti’s criteria (only LGG), and methylation of the O(6)-Methylguanine-DNA methyltransferase promotor (only HGG)(Supplementary Figure 1). Pignatti’s criteria are unfavorable prognostic factors for survival in patients with LGG.^17^ Potential follow-up treatment as showed in Supplementary Figure 1 for all different grades included: TMZ-chemoradiation (30 fractions radiation, concurrent Temozolomide 75 mg/m^2^ (TMZ) daily, adjuvant TMZ in 12 cycles 200 mg/m^2^ days 1–5 q4wk), hypo-TMZ-chemoradiation (15 fractions radiation, concurrent 75 mg/m^2^ TMZ daily, adjuvant TMZ six cycles 200 mg/m^2^ days 1–5 q4wk), sequential radio- and chemotherapy (30 fractions radiotherapy, followed by either 12 cycles TMZ 200 mg/m^2^ days 1–5 q4wk or 6 cycles Lomustine 110 mg/m^2^ day 1, Procarbazine 60 mg/m^2^ days 8–21, Vincristine 2 mg days 8 and 29 q6wk (PCV) chemotherapy), adjuvant radiotherapy (60 Gy/30 fractions), adjuvant hypo-fractionated radiotherapy (52.5 Gy/15 fractions), adjuvant 12 cycles TMZ 200 mg/m^2^ days 1–5 q4wk monotherapy, adjuvant TMZ “elderly” (6 cycles 100 mg/m^2^ days 1–7 and 15–21 q4wk, adjusted dosing schedule), Best Supportive Care (BSC), and experimental (includes any form of clinical experimental treatment for diffuse glioma conducted within the UMCU, which either replaces or supplements conventional treatment). The blinded assessment of optimal treatment according to the treatment guideline was compared to the initiated treatment during the first postoperative patient consultation; initiated treatment was then classified as “undertreatment” or not. For example, a patient is eligible for “TMZ-chemoradiation” therapy according to the flowchart. However, after surgery, after discussion in the multidisciplinary tumor board and discussion with the patient, the treating physician initiates TMZ monotherapy. This is an example of a reduction in treatment intensity compared to treatment-per-guideline, referred to as undertreatment. Of note, data from neuropsychological assessment were not discussed in detail during postoperative decision making with the tumor board, although they sometimes were available in the patient’s electronical file. In general, the global cognitive performance of a patient was included when discussing the overall functional status of the patient, without having formal status.

Because changes in state-of-the-art-therapy over time, patients might be incorrectly labeled as “undertreated”, especially patients who were included at the beginning of our cohort. We, therefore, studied the subgroups in which this could be the case:

- Grade III Astrocytoma IDH-mutated from 2010 to 2016. From 2016 these patients received TMZ in addition to radiotherapy. So when patients had received radiotherapy monotherapy between 2010 and 2016 they could have been wrongly classified as undertreated based on the most recent guidelines wherein radiotherapy + TMZ was the new standard-of-care.
- Grade III Oligodendroglioma 1p19q deletion from 2010 to 2012. After 2012 these patients received PCV in addition to radiotherapy according to most recent guidelines. In this way, when patients had received radiotherapy monotherapy between 2010 and 2012 they could have been incorrectly labeled as “undertreated”.

In these specific subgroups, we investigated when “undertreatment” was scored. If so, we checked in the electronic patient file whether this was the case because the standard treatment changed over time. In other words, whether a patient was actually not undertreated regarding to the state-of the-art-therapy within that specific time period.

To test compliance, we evaluated the percentage of administered follow-up treatment, and potential dose reduction during treatment. The percentage of administered treatment was calculated as the fraction of the total planned treatment that the patient actually received. We determined this percentage for postoperative radio- and chemotherapy. For example, a patient should have received 30 fractions of radiotherapy. Eventually, this patient only received 15 fractions because he refused further therapy. The percentage of administered treatment is 50%. Dose reduction was evaluated based on reports of treating physicians in the electronic patient files.

To assess complications during follow-up, all complications between surgery and last day of follow-up treatment were extracted from electronic patients’ files. Complications were defined as every unfavorable sign, symptom, or disease, which might be considered as related to the treatment. Of note, causality did not have to be ascertained, as this is often impossible. Complications were divided in nine main groups: general, infectious, coagulation, neurological (central and peripheral), gastrointestinal, skin, hematological, and other causes of complications. Supplementary Table 1 shows classification of various complications within each main group. The severity of all complications was assessed based on the Common Terminology Criteria for Adverse Events (CTCAE) protocol (v4.3) of the National Cancer Institute.^18^ Complication severity was dichotomized into “mild-moderate” (score 1–2) and “severe” (score ≥3). In our analyses, we finally used “severe complication” as intermediate factor, defined as “Common Terminology Criteria for Adverse Events score of ≥3”.

### Statistical Analyses

Analyses were performed with RStudio (v1.1.463). We analyzed baseline characteristics with descriptive statistics (Table 1). In order to avoid bias due to missing data, we imputed missing values for all variables by means of multiple imputation, through the R-package “Mice” impute() function for random missing values.

**Table 1:** Demographics of the Total Cohort, as well as the LGG and HGG Cohort Separately

	LGG patients, *n* (%)	HGG patients, *n* (%)	Total cohort, *n* (%)
N	111	143	254
Gender, female	40 (36.0%)	49 (34.3%)	89 (35.0%)
Age at first surgery (median [IQR])	*42 [38–52]*	*63 [54–68]*	*55 [41–65]*
Karnofsky Performance Status ≥70	109 (98.2%)	128 (89.5%)	237 (93.3%)
WHO 2016 grade			
Grade II/III astrocytoma IDH-M	64 (57.7%)	NA	64 (25.2%)
Grade II/III oligodendroglioma IDH-M 1p19q	47 (42.3%)	NA	47 (18.5%)
Grade II/III astrocytoma IDH-WT	NA	13 (9.1%)	13 (5.1%)
Grade IV glioblastoma IDH-M	NA	7 (4.9%)	7 (2.8%)
Grade IV glioblastoma IDH-WT	NA	123 (86.0%)	123 (48.4%)
Tumor volume (cm^3^)*(median [IQR])*	*51.43 [20.64–75.91]*	*63.98 [27.73–132.50]*	*56.64 [22.23–105.36]*
Extent of resection			
1–78%	53 (47.7%)	30 (21.0%)	83 (32.7%)
79–90%	20 (18.0%)	32 (22.4%)	52 (20.5%)
91–100%	17 (15.3%)	62 (43.4%)	79 (31.1%)
Not reported	21 (18.9%)	19 (13.3%)	40 (15.7%)
Location (measured on T2 FLAIR)			
Frontal	93 (83.8%)	115 (80.4%)	208 (81.9%)
Temporal	45 (40.5%)	83 (58.0%)	128 (50.4%)
Parietal	32 (28.8%)	81 (56.6%)	113 (44.5%)
Occipital	9 (8.1%)	31 (21.7%)	40 (15.7%)
Hemiphere			
Left	66 (59.5%)	99 (69.2%)	165 (65.0%)
Right	39 (35.1%)	36 (25.2%)	75 (29.5%)
Both	6 (5.4%)	8 (5.6%)	14 (5.5%)
Epilepsy at presentation	80 (72.1%)	77 (53.8%)	157 (61.8%)
Neurocognitive functioning*(threshold)*	*≤–1.5 SD*	*≤–2.0 SD*	*≤–1.5 SD/ ≤–2.0 SD*
Attention and executive functioning	37 (33.3%)	50 (35.0%)	116 (45.7%)/ 67 (26.4%)
Memory	25 (22.5%)	54 (37.8%)	108 (42.5%)/ 59 (23.2%)
Language	11 (9.9%)	31 (21.7%)	56 (22.0%)/ 36 (14.2%)
Visuospatial functioning	16 (14.4%)	36 (25.2%)	68 (26.8%)/ 44 (17.3%)
Psychomotor speed	19 (17.1%)	39 (27.3%)	70 (27.6%)/ 52 (20.5%)
Total impaired domains*(median [IQR])*	*0 [0–1]*	*1 [0–2]*	*1 [0–3]/ 0 [0–2]*
Treatment			
Radiotherapy	52 (46.8%)	124 (86.7%)	176 (69.3%)
Chemotherapy	41 (36.9%)	115 (80.4%)	156 (61.4%)
Experimental^a^/(hypo-^b^)TMZ-chemoradiation^c^	6 (5.4%)	117 (81.8%)	123 (48.4%)
Radiotherapy + 12 cycles TMZ	13 (11.7%)	3 (2.1%)	16 (6.3%)
Radiotherapy + 6 cycles PCV	21 (18.9%)	1 (0.7%)	22 (8.7%)
Standard intensity monotherapy^d^	20 (18.0%)	20 (14.0%)	40 (15.7%)
6 cycles TMZ (“elderly”)	0 (0.0%)	2 (1.4%)	2 (0.8%)
Wait and scan	51 (45.9%)	7 (4.9%)	58 (22.8%)
Best supportive care	0 (0.0%)	1 (0.7%)	1 (0.4%)
Undertreatment			
Initiated treatment differed from guideline	38 (34.2%)	27 (18.9%)	65 (25.6%)
Initiated treatment lesser intensity than guideline	22 (19.8%)	17 (11.9%)	39 (15.4%)
Compliance			
%-radiotherapy received (*median [IQR])*	*100 [100–100]*	*100 [100–100]*	*100 [100–100]*
Receiving <100% radiotherapy	0 (0%)	2 (1.6%)	2 (1.1%)
%-chemotherapy received*(median [IQR])*	*100 [85.7–100]*	*100 [100–100]*	*100 [90.2–100]*
Duration of chemotherapy<100%	13 (31.7%)	28 (24.3%)	41 (26.3%)
Dose reduction chemotherapy	18 (16.2%)	36 (25.2)	54 (21.3)
Complication, according to CTCAE			
Number of complications*(median [IQR])*	*4 [2–7]*	*7 [5–10]*	*6 [3–9]*
Grade 1	99 (89.2%)	134 (93.7%)	233 (91.7%)
Grade 2	70 (63.1%)	122 (85.3%)	192 (75.6%)
≥ Grade 3	23 (20.7%)	65 (45.5%)	88 (34.6%)
Survival, days*(median [IQR])*	*1778 [919–2702]*	*539 [303–848]*	*809 [465–1803]*

LGG corresponds to grade II/III astrocytoma IDH-mutated and grade II/II oligodendroglioma 1p19q deletion. HGG corresponds to grade II/III astrocytoma IDH-wildtype, glioblastoma IDH-mutated and IDH-wildtype. Variables are shown in number with valid percentages or median with interquartile range (IQR). IDH, isocitrate dehydrogenase; M, mutant; 1p19q, 1p19q codeletion; WT, wild type; TMZ, Temozolomide; PCV, Procarbazine, Lomustine, Vincristine; CTCAE, Common Terminology Criteria for Adverse Events; LGG, low-grade glioma; HGG, high-grade glioma; NA, not applicable.

^a^Any form of clinical experimental treatment for diffuse glioma conducted within the UMCU, including experimental immunotherapy, which either replaces or supplements conventional treatment

^b^15 fractions radiation, concurrent Temozolomide daily, and adjuvant 12 cycles Temozolomide

^c^30 fractions radiation, concurrent Temozolomide daily and adjuvant 12 cycles Temozolomide

^d^Monotherapy consisted of one of the following: 30 fractions radiotherapy, 12 cycles Temozolomide, or 6 cycles Procarbazine, Lomustine, and Vincristine

Since the various glioma subtypes differ greatly in their biological behavior as well as their prognosis, it is possible that the effect of cognition—and other determinants—on survival also differs between WHO 2016 glioma subtypes. For this reason, we performed all analyses in subgroups of higher grade (Grade II/III Astrocytoma IDH-Wildtype, Glioblastoma IDH-mutated, and IDH-Wildtype) and lower grade glioma (Grade II/III Astrocytoma IDH-mutated, Grade II/III Oligodendroglioma 1p19q deletion).

To test our hypothesis regarding possible intermediate factors, we used Structural Equation Modeling^19^ to perform mediation analyses. Mediation analyses are analyses to evaluate whether a variable is a factor *in* the causal chain, referred to as an intermediate factor. Intermediate factors are different in that way from confounders. A confounder is a third factor that affects both the determinant and outcome, implying these variables to be (partially) related when they are, in fact, not. In contrast, a mediator is a factor *between* the determinant and outcome, explaining the process by which the determinant and outcome are related. This is an important difference, because generally results are not corrected for intermediate factors, but they are for confounders. Mediation analyses were carried out with the “sem” function of the Lavaan package in R.^19^ The lavaan package is developed to estimate a large variety of multivariate statistical models, including structural equation modeling.

**Fig. 1:**
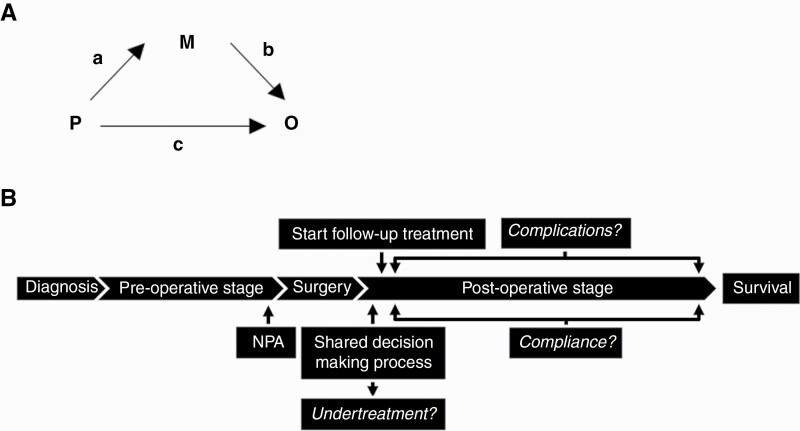
(A) Principle of mediation analyses. P, predictor/exposure variable; M, mediator variable; O, outcome variable; a * b, indirect effect; c, direct effect; c + (a * b), total effect. (B) Simplified timeframe in the order in which the studied events take place and mediators are determined. NPA, neuropsychological assessment.

Figure 1A shows the principle of mediation analyses. A simplified timeframe in the order in which the studied events take place and mediators are determined is presented in Figure 1B. The specific paths are indicated by the letters “a”, “b”, and “c”, wherein “a” is the path between determinant (P) and mediator (M), “b” is the path between the mediator (M) and outcome (O), and “c” is the path between determinant (P) and outcome (O). A specific variable can be considered as a mediator between determinant and outcome if the indirect effect (a * b) and the total effect (c + (a * b)) are significant paths, while the direct effect (c) is not. As predictor variables (P) we used number of neurocognitive deficits in all five domains and neurocognitive deficits in each of the five domains separately. Mediator variables (M) were grouped into “undertreatment”, “compliance”, and “complication”. Only 2 patients received less than 100% radiotherapy of their initially planned radiotherapy dose. Because of this minimal number of events, “percentage received radiotherapy” was not included as a mediator variable. All mediators were separately included in four models. One model tested “undertreatment” (received treatment intensity versus planned intensity), one model tested “complication” (presence of CTCAE ≥3), and two models tested “compliance” (percentage chemotherapy received of total planned, and dose reduction). As outcome variable (O), we used 1.5-year overall survival for high-grade glioma (HGG) patients, and 5-years overall survival for LGG patients. The survival outcome measure was based on the data-driven median split of survival days for each group separately. Various determinants were included in the mediation model as confounders. Supplementary Table 2 shows all covariates included in the model, per glioma subgroup. In Supplementary Figure 2, we show all the possible relations between covariates, determinant, and outcome that were considered for our analyses in a directed acyclic graph^20^ Before performing mediation analyses, we tested for multicollinearity between all covariates included in the mediation models by visualizing determinants in scatterplots and by Pearson correlation coefficients. An R of >0.4 was considered as collinear. Moreover, the associations between cognition (determinant) and undertreatment, compliance, and complication (mediator variables) were evaluated by means of univariable logistic regression analyses for binary outcome measures (undertreatment, complication, and dose reduction) and linear regression analyses for continuous outcome measures (percentage chemotherapy received). We also carried out multivariable logistic and linear regression analyses between cognition and all mediator variables, adjusting for all covariates reported in Supplementary Table 2. A commonly applied general rule for sample size is 15 cases per predictor in a standard ordinary least squares multiple regression analysis. Since SEM is closely related to multiple regression in some respects, it is defendable to approximately include 15 cases per measured variable.^21^

Of each mediation model, we reported effect sizes with 95% confidence intervals and p-values of the direct and indirect effects, as well as the total effect. The direct effect is path “c” from the predictor variable (P) to the outcome variable (O) (Figure 1); that is, in our analyses, the direct effect of cognition on survival, without any mediator. The indirect effect (a * b) is path “a” from the predictor variable (P) to the mediator variable (M) *plus* path “b” from the mediator variable (M) to the outcome variable (O) (Figure 1). For example, path “a” is the effect of cognition on the presence of severe complications, and path “b” is the effect of the presence of severe complications on survival. The total effect is calculated by c + (a * b). So, in this example, the complete effect of cognition on survival, together with the presence of severe complications as potential mediator. If both the total effect and the direct effect are significant, while the indirect effect is not, the effect of cognition on survival is *not* explained by the presence of a mediating factor, in this case severe complications. Should the total effect and the indirect effect be significant, while the direct effect is not, then the effect of cognition on survival is explained by the presence of severe complications. All pathways are corrected for the confounders listed in Supplementary Table 2. The estimator provided by Lavaan is the diagonally weighted least squares (DWLS), which is used for dichotomous outcome measures in structural equation modeling.^22^*P*-values ≤ .05 were considered significant.

## Results

### Baseline Patient Characteristics

In total 254 patients were included, of whom 111 patients were LGG patients and 143 were HGG patients. Demographics of the total cohort, as well as the LGG and HGG cohort separately, are shown in Table 1. Age ranged from 19 to 82 years old, with a median age of 55 years old. Tumor volume ranged from 1.0 to 277.8 cm^3^, with a median of 56.6 cm^3^. The most frequently impaired domain was memory (37.8% ≤–2 SD) in HGG and attention and executive functioning in LGG (33.3≤–1.5 SD). Neuropsychological assessment data was missing in 14.2% and 8.3% of patients for domains visuospatial functioning and language, respectively. Other domains had missing values in 2.0–5.5% of patients. Regarding mediator and confounding variables, KPS had most missing data, corresponding to 5.9% of patients. Other mediator variables and confounders has missing data in 1.6%-4.3% of patients.

### Follow-up

Follow-up data are shown in Table 1 as well. Of all patients, 176 patients received *postoperative* radiotherapy and 156 patients received chemotherapy. TMZ-chemoradiation was initiated in 114 patients. Monotherapy radiotherapy or chemotherapy was given in 40 patients. Monotherapy chemotherapy included TMZ, TMZ elderly or PCV monotherapy. Fifty-eight LGG patients followed a “wait & scan” policy. Subsequent survival ranged from 0 to 29.4 years, with a median survival of 2.2 years. 1.5-year survival was 48.3% in HGG patients and 91.9% in LGG patients. 5-years survival was 7.7% in HGG patients and 46.8% in LGG patients.

### Correlation of Cognition With Intermediate Factors

Results of univariable and multivariable analyses of cognition on all intermediate factors are shown in Table 2. In HGG patients, impaired visuospatial functioning was associated with an increased risk of undertreatment, defined as initiated treatment of less intensity compared to treatment-per-guideline. Moreover, impairments in the domain language were associated with an increased risk of severe complications. Regarding patients’ compliance, total number of impaired domains as well as the domain language appeared to be negatively associated with the percentage of received chemotherapy (compared to initiated chemotherapy dose) in HGG patients. After adjusting for confounders, we still found an increased risk of undertreatment in patients with HGG and impaired visuospatial functioning, as well as an increased risk of non-compliance in HGG patients with impairments in the domain language and with multiple impaired domains.

**Table 2:** Results of Univariable and Multivariable Regression Analyses of Association of Neurocognitive Deficits With Undertreatment, Complication, and Compliance

	Univariable analyses				Multivariable analyses			
Neurocognitive deficit	LGG		HGG		LGG^a^		HGG^b^	
Undertreatment	*OR (95%-CI)*	*P-*value	*OR (CI 95%)*	*P-value*	*OR (CI 95%)*	*P-value*	*OR (CI 95%)*	*P-value*
Total impaired domains, ref = 0		.579^c^		.998^c^		.629^c^		.988^c^
1 impaired domain	0.321 (0.085–1.222)	.096	0.793 (0.213–2.945)	.729	0.340 (0.085–1.352)	.125	0.617 (0.140–2.717)	.523
2 impaired domains	1.500 (0.391–5.752)	.554	0.838 (0.197–3.560)	.810	1.656 (0.399–6.871)	.487	1.045 (0.202–5.400)	.958
3 impaired domains	1.857 * 10^–9^ (0.000–∞)	.999	0.683 (0.129–3.608)	.653	2.359 * 10^–9^ (0.000–∞)	.999	0.739 (0.121–4.496)	.742
4 impaired domains	0.600 (0.064–5.583)	.654	0.683 (0.074–6.253)	.735	0.985 (0.086–11.238)	.991	0.653 (0.061–6.995)	.724
5 impaired domains	1.857 * 10^–9^ (0.000–∞)	1.000	3.803 * 10^–9^ (0.000–∞)	.999	1.169 * 10^–9^ (0.000–∞)	1.000	8.571 * 10^–9^ (0.000–∞)	.999
Attention and EF	0.702 (0.249–1.974)	.502	0.360 (0.098–1.319)	.123	0.788 (0.259–2.402)	.676	0.409 (0.105–1.600)	.199
**Complication**	*OR (CI 95%)*	*P-value*	*OR (CI 95%)*	*P-value*	*OR (CI 95%)*	*P-value*	*OR (CI 95%)*	*P-value*
Total impaired domains, ref = 0		.718^c^		.447^c^		.949^c^		.671^c^
1 impaired domain	0.982 (0.324–2.974)	.974	1.497 (0.620–3.612)	.369	0.682 (0.205–2.270)	.533	1.245 (0.484–3.203)	.649
2 impaired domains	0.818 (0.156–4.282)	.812	1.641 (0.619–4.347)	.319	0.596 (0.101–3.530)	.568	1.418 (0.496–4.056)	.515
3 impaired domains	1.023 (0.104–10.082)	.985	1.778 (0.622–5.078)	.283	0.621 (0.057–6.827)	.697	1.376 (0.454–4.173)	.573
4 impaired domains	4.091 (0.725–23.093)	.111	4.148 (0.953–18.052)	.058	1.625 (0.205–12.867)	.645	3.686 (0.797–17.049)	.095
5 impaired domains	2.532 * 10^–9^ (0.000–∞)	1.000	3.556 (0.301–41.991)	.314	2.573 * 10^–9^ (0.000–∞)	1.000	2.742 (0.212–35.510)	.440
Attention and EF	1.378 (0.533–3.562)	.509	1.500 (0.752–2.993)	.250	0.785 (0.260–2.370)	.668	1.372 (0.661–2.847)	.396
**Compliance—dichotomous**	*OR (CI 95%)*	*P-value*	*OR (CI 95%)*	*P-value*	*OR (CI 95%)*	*P-value*	*OR (CI 95%)*	*P-value*
Total impaired domains, ref = 0		.835^c^		.916^c^		.782^c^		.871^c^
1 impaired domain	1.262 (0.498–3.199)	.624	0.890 (0.335–2.366)	.815	1.073 (0.409–2.812)	.886	1.085 (0.371–3.172)	.882
2 impaired domains	2.294 (0.646–8.143)	.199	1.446 (0.520–4.026)	.480	2.264 (0.621–8.258)	.216	1.700 (0.546–5.296)	.360
3 impaired domains	0.574 (0.060–5.519)	.630	1.385 (0.458–4.189)	.564	0.431 (0.042–4.393)	.477	1.693 (0.506–5.665)	.393
4 impaired domains	1.147 (0.191–6.873)	.881	1.714 (0.419–7.006)	.453	0.683 (0.095–4.889)	.704	2.146 (0.469–9.827)	.325
5 impaired domains	1.420 * 10^–9^ (0.000–∞)	1.000	1.592 * 10^–9^ (0.000–∞)	.999	9.205 * 10^–10^ (0.000–∞)	1.000	1.751 * 10^–9^ (0.000–∞)	.999
Psychomotor speed	1.206 (0.431–3.374)	.722	1.448 (0.662–3.166)	.353	0.929 (0.299–2.883)	.899	1.875 (0.791–4.446)	.153
Visuospatial functioning	0.626 (0.187–2.096)	.448	1.712 (0.774–3.787)	.185	0.428 (0.114–1.600)	.207	1.904 (0.804–4.506)	.143
Language	1.160 (0.317–4.245)	.822	0.616 (0.243–1.562)	.307	1.075 (0.282–4.095)	.916	0.648 (0.239–1.755)	.394
Memory	0.560 (0.202–1.551)	.265	0.456 (0.207–1.005)	.052	0.459 (0.157–1.341)	.155	0.478 (0.206–1.111)	.086
Attention and EF	0.941 (0.406–2.180)	.887	0.632 (0.290–1.377)	.248	0.729 (0.290–1.816)	.493	0.603 (0.262–1.388)	.234
**Compliance—continuous**	*Bèta (CI 95%)*	*P-value*	*Bèta (CI 95%)*	*P-value*	*Bèta (CI 95%)*	*P-value*	*Bèta (CI 95%)*	*P-value*
Total impaired domains	–0.269 (–3.779 to 3.240)	.879	–3.566 (–6.243 to –0.889)	.009 *	–0.247 (–3.999 to 3.506)	.897	–3.448 (–6.308 to –0.587)	.019 *
Psychomotor speed	–1.695 (–12.763 to 9.372)	.762	–6.601 (–14.994 to 1.792)	.122	–2.785 (–14.864 to 9.294)	.648	–6.276 (–15.143 to 2.590)	.164
Visuospatial functioning	–5.408 (–17.237 to 6.421)	.367	–6.491 (–15.109 to 2.128)	.139	–6.420 (–19.027 to 6.187)	.315	–4.873 (–13.945 to 4.198)	.290
Language	–0.460 (–14.417 to 13.498)	.948	–14.588 (–23.409 to –5.767)	.001 *	–1.890 (–16.254 to 12.474)	.795	–14.878 (–24.104 to –5.653)	.002 *
Memory	1.544 (–8.435 to 11.523)	.760	–5.430 (–13.154 to 2.294)	.167	0.824 (–9.557 to 11.204)	.875	–4.857 (–13.129 to 3.415)	.248
Attention and EF	2.536 (–6.297 to 11.370)	.570	–2.322 (–10.218 to 5.574)	.562	2.560 (–7.000 to 12.121)	.597	–2.084 (–10.334 to 6.166)	.618

Determinants were total impaired neurocognitive domains and all domains separately at ≤–1.5 in low-grade glioma (LGG) patients, and at ≤–2.0 in high-grade glioma (HGG) patients. LGG corresponds to grade II/III astrocytoma IDH-mutated and grade II/II oligodendroglioma 1p19q deletion. HGG corresponds to grade II/III astrocytoma IDH-wildtype, glioblastoma IDH-mutated and IDH-wildtype. As outcome variables, decreased intensity of planned treatment compared to guidelines was used for “undertreatment”, presence of severe complication based on the CTCAE protocol was used for “complication”, dose reduction of chemotherapy was used for ‘compliance—dichotomous, and percentage of received chemotherapy of planned was used for “compliance—continuous”. Significant *P*-values (≤.05) are marked with an asterisk (*). EF, executive functioning, OR, odds ratio, CI, confidence interval.

^a^ Results of multivariable analyses in the LGG subgroup were adjusted for age at awake surgery, preoperative tumor volume, Karnofsky performance scale (≥70 or <70) and WHO 2016 glioma classification.

^b^ Results of multivariable analyses in the HGG subgroup were adjusted for age at awake surgery, preoperative tumor volume, Karnofsky performance scale (≥70 or <70), WHO 2016 glioma classification and epilepsy at presentation.

^c^ *P*-value of the entire variable “total impaired domains”

### Undertreatment Does Not Explain the Relation Between Cognition and Survival

In 26.4% of patients, the initiated treatment was different from the treatment-per-guideline. In 19.8% of the LGG patients and 11.9% of the HGG patients the initiated treatment concerned a treatment of decreased intensity compared to treatment-per-guideline (Table 1). Results of mediation analyses are shown in Table 3. In HGG patients, total number of impaired domains and the domain memory showed significant correlations with survival, which could not be explained by undertreatment as mediator. Specifically, the direct effects, as well as the total effects of total number of impaired domains and the domain memory on survival, were significant (*P* < .001 and *P* < .001, respectively), while the indirect effect including the intermediate factor undertreatment were not significant (*P* = .657). In LGG patients, total number of impaired domains and the domains language and attention and executive functioning were negatively correlated with 5-year survival (*P* = .032, *P* = .029, and *P* < .001, respectively). These results could not be explained by a direct nor indirect effect with undertreatment as intermediator factor, since both the indirect (a*b) and the direct paths (c) were not significantly associated with survival in these domains (indirect: *P* = .933, *P* = .519, and *P* = .583, respectively; direct: *P* = .381, *P* = .688, and *P* = .095, respectively). We may conclude from this analysis that undertreatment was not a mediator between these cognitive domains and survival. However, the direct effect of these domains on survival did not appear to be significant either. This suggests that another unknown mechanism caused the overall significant negative effect of these impaired cognitive domains on survival.

**Table 3:** Results of Mediation Analyses for “Undertreatment”

Neurocognitive deficit per domain		LGG		HGG	
Undertreatment		*est DWLS (95%-CI)*	*P-value*	*est DWLS (95%-CI)*	*P-value*
Total impaired domains	Direct (c)	2.069 (–2.564 to 6.702)	.381	0.781 (0.647–0.915)	<.001*
	Indirect (a * b)	–0.141 (–3.415 to 3.132)	.933	–0.015 (–0.082 to 0.052)	.657
	Total (c + (a * b)	1.928 (0.166–3.690)	.032*	0.766 (0.651–0.880)	<.001*
Psychomotor speed	Direct (c)	0.073 (–0.591 to 0.738)	.829	0.065 (–0.210 to 0.340)	.643
	Indirect (a * b)	0.219 (–0.142 to 0.581)	.235	0.005 (–0.035 to 0.045)	.808
	Total (c + (a * b)	0.292 (–0.084 to 0.668)	.127	0.070 (–0.205 to 0.345)	.618
Visuospatial functioning	Direct (c)	0.269 (–9.742 to 10.279)	.958	–0.161 (–0.550 to 0.228)	.417
	Indirect (a * b)	–0.407 (–10.742 to 9.928)	.938	0.063 (–0.125 to 0.250)	.513
	Total (c + (a * b)	–0.139 (–0.541 to 0.264)	.499	–0.099 (–0.409 to 0.212)	.534
Language	Direct (c)	0.175 (–0.680 to 1.030)	.688	0.240 (–0.120 to 0.599)	.191
	Indirect (a * b)	0.196 (–0.400 to 0.792)	.519	–0.049 (–0.182 to 0.083)	.465
	Total (c + (a * b)	0.371 (0.039–0.703)	.029*	0.190 (–0.129 to 0.510)	.242
Memory	Direct (c)	0.009 (–3.669 to 3.686)	.996	0.417 (0.177–0.658)	.001*
	Indirect (a * b)	0.265 (–3.164 to 3.695)	.880	0.004 (–0.030 to 0.038)	.817
	Total (c + (a * b)	0.274 (–0.064 to 0.612)	.112	0.422 (0.182–0.661)	.001*
Attention and executive functioning	Direct (c)	0.477 (–0.083 to 1.037)	.095	0.245 (–0.071 to 0.560)	.128
	Indirect (a * b)	0.106 (–0.272 to 0.483)	.583	–0.065 (–0.225 to 0.095)	.424
	Total (c + (a * b)	0.583 (0.319–0.847)	<.001*	0.180 (–0.085 to 0.445)	.184

Predictor variable (neurocognitive domains) are shown on the left, divided into three effect estimates: direct (c), indirect (a * b) and total effect (c + (a * b)). See Figure 1 for further explanation of these effects. Included mediator is “Intensity of initiated treatment versus optimal treatment-per-guideline”, classified as score 0 in case intensity was equal to or higher than treatment-per-guideline and score 1 when intensity was less than treatment-per-guideline. Analyses were performed in low-grade glioma (LGG) and high-grade glioma (HGG) subgroups separately. LGG corresponds to grade II/III astrocytoma IDH-mutated and grade II/II oligodendroglioma 1p19q deletion. HGG corresponds to grade II/III astrocytoma IDH-wildtype, glioblastoma IDH-mutated and IDH-wildtype. Estimates are reported as diagonally weighted least squares (est DWLS) with 95% confidence interval (CI) and *P*-value. Significant *P*-values (≤.05) are marked with an asterisk(*).

The specific subgroups we studied showed only one case wherein we incorrectly labeled a patient as “undertreated” due to changes in state-of-the-art-therapy over time.

### Compliance Does Not Mediate the Correlation Between Cognition and Survival

Of all included patients, 54 patients had a dose reduction of chemotherapy, of whom were 18 LGG patients and 36 HGG patients (Table 1). As reported in the methods section, “percentage received radiotherapy” was not included as possible mediator in mediation analyses because of a minimal number of events. Of 156 patients who received chemotherapy, 115 received full-dose chemotherapy (100% of initiated treatment), corresponding to 73.7% of patients.

Results of mediation analyses for “compliance” are reported in Table 4. Results of the first analysis, including dose reduction as potential intermediate factor, showed a significant association between the domain memory and 1.5-years survival of HGG patients (*P* = .011). The effect of memory on survival was not mediated by dose reduction, since the indirect effect (a*b) was not significant (*P* = .997), while the direct effect (c) was (*P* = .023). The second analysis, including percentage received chemotherapy as potential intermediate factor, showed similar results. Both the total and direct effects of memory on 1.5-years survival were significant (*P* = .009 and *P* = .036, respectively), and the indirect effect was not significant (*P* = .259). In other words, the effect of the domain memory on survival is not mediated by the percentage of received chemotherapy. Moreover, the second analysis showed that in LGG patients the total number of impaired domains was significantly associated with a decreased 5-years survival via a direct effect (c; *P* < .001), rather than via an indirect effect (a*b; *P* = .331) with percentage of received chemotherapy as mediator. So, the percentage of received chemotherapy did not mediate the effect of cognition on 5-year survival in LGG patients.

**Table 4:** Results of Mediation Analyses for “Compliance”

Neurocognitive deficit per domain		LGG		HGG	
Compliance—dichotomous		*est DWLS (95%-CI)*	*P-value*	*est DWLS (95%-CI)*	*P-value*
Total impaired domains	Direct (c)	–0.370 (–2.080 to 1.340)	.672	0.087 (–0.078 to 0.252)	.302
	Indirect (a * b)	0.464 (–1.590 to 2.518)	.658	–0.004 (–0.026 to 0.018)	.749
	Total (c + (a * b)	0.094 (–0.446 to 0.634)	.733	0.083 (–0.086 to 0.253)	.335
Psychomotor speed	Direct (c)	–0.097 (–0.995 to 0.801)	.832	0.088 (–0.197 to 0.373)	.544
	Indirect (a * b)	0.053 (–0.095 to 0.201)	.483	–0.017 (–0.069 to 0.036)	.533
	Total (c + (a * b)	–0.044 (–0.945 to 0.858)	.924	0.072 (–0.211 to 0.354)	.619
Visuospatial functioning	Direct (c)	–0.682 (–1.561 to 0.196)	.128	–0.007 (–0.355 to 0.342)	.969
	Indirect (a * b)	0.422 (–0.003 to 0.847)	.052	–0.024 (–0.098 to 0.049)	.518
	Total (c + (a * b)	–0.261 (–0.982 to 0.460)	.478	–0.031 (–0.359 to 0.296)	.852
Language	Direct (c)	–0.500 (–1.364 to 0.363)	.256	0.266 (–0.071 to 0.602)	.122
	Indirect (a * b)	0.254 (–0.011 to 0.519)	.061	0.012 (–0.040 to 0.064)	.647
	Total (c + (a * b)	–0.246 (–1.104 to 0.611)	.574	0.278 (–0.048 to 0.603)	.095
Memory	Direct (c)	–0.127 (–1.346 to 1.093)	.839	0.343 (0.048–0.637)	.023*
	Indirect (a * b)	0.244 (–0.073 to 0.561)	.131	0.000 (–0.115 to 0.115)	.997
	Total (c + (a * b)	0.117 (–0.956 to 1.190)	.830	0.342 (0.078–0.607)	.011*
Attention and executive functioning	Direct (c)	0.281 (–0.413 to 0.976)	.427	0.080 (–0.227 to 0.387)	.609
	Indirect (a * b)	0.046 (–0.100 to 0.192)	.536	0.022 (–0.053 to 0.098)	.562
	Total (c + (a * b)	0.327 (–0.370 to 1.025)	.358	0.103 (–0.188 to 0.393)	.488
**Compliance to continuous**		*est DWLS (95%-CI)*	*P-value*	*est DWLS (95%-CI)*	*P-value*
Total impaired domains	Direct (c)	0.673 (0.449–0.898)	<.001*	–0.029 (–0.237 to 0.180)	.787
	Indirect (a * b)	0.038 (–0.038 to 0.113)	.331	0.103 (–0.008 to 0.215)	.069
	Total (c + (a * b)	0.711 (0.479–0.942)	<.001*	0.074 (–0.098 to 0.247)	.399
Psychomotor speed	Direct (c)	–14.554 (–53.963 to 24.855)	.469	0.003 (–0.288 to 0.293)	.987
	Indirect (a * b)	14.511 (–24.208 to 53.230)	.463	0.075 (–0.043 to 0.194)	.213
	Total (c + (a * b)	–0.043 (–0.937 to 0.851)	.925	0.078 (–0.206 to 0.362)	.592
Visuospatial functioning	Direct (c)	–38.236 (–148.525 to 72.052)	.497	–16.226 (–43.526 to 11.073)	.244
	Indirect (a * b)	37.978 (–72.014 to 147.970)	.499	16.204 (–10.998 to 43.407)	.243
	Total (c + (a * b)	–0.258 (–0.975 to 0.458)	.480	–0.022 (–0.349 to 0.305)	.895
Language	Direct (c)	–0.311 (–1.063 to 0.441)	.417	0.114 (–0.246 to 0.475)	.535
	Indirect (a * b)	0.066 (–0.116 to 0.248)	.479	0.144 (–0.015 to 0.304)	.076
	Total (c + (a * b)	–0.245 (–1.099 to 0.608)	.573	0.259 (–0.070 to 0.588)	.123
Memory	Direct (c)	–16.208 (–95.857 to 63.441)	.690	0.292 (0.018–0.566)	.036*
	Indirect (a * b)	16.322 (–62.584 to 95.228)	.685	0.059 (–0.043 to 0.161)	.259
	Total (c + (a * b)	0.114 (–0.953 to 1.181)	.834	0.351 (0.086–0.616)	.009*
Attention and executive functioning	Direct (c)	–10.727 (–85.786 to 64.332)	.779	0.088 (–0.196 to 0.371)	.545
	Indirect (a * b)	11.049 (–63.476 to 85.574)	.771	0.024 (–0.069 to 0.116)	.617
	Total (c + (a * b)	0.322 (–0.371 to 1.014)	.363	0.111 (–0.181 to 0.403)	.455

Predictor variable (neurocognitive domains) are shown on the left, divided into three effect estimates: direct (c), indirect (a * b) and total effect (c + (a * b)). See Figure 1 for further explanation of these effects. Included mediators are dose reduction of chemotherapy (compliance—dichotomous) and percentage received chemotherapy dose of total planned dose (compliance—continuous). Analyses were performed in low-grade glioma (LGG) and high-grade glioma (HGG) subgroups separately. LGG corresponds to grade II/III astrocytoma IDH-mutated and grade II/II oligodendroglioma 1p19q deletion. HGG corresponds to grade II/III astrocytoma IDH-wildtype, glioblastoma IDH-mutated and IDH-wildtype. Estimates are reported as diagonally weighted least squares (est DWLS) with 95% confidence interval (CI) and *P*-value. Significant *P*-values (≤.05) are marked with an asterisk (*).

### Complication is Not an Intermediate Factor Between Cognition and Survival

In the LGG subgroup, 23 patients had a severe complication (CTCAE 3 or more). Of HGG patients, 65 had a severe complication (Table 1). Results from mediation analyses for “complication” are shown in Table 5. In contrast to the indirect effect, the direct and total effect of total impaired domains on survival were significant in both subgroups (LGG: *P* = .023 and *P* = .016, respectively; HGG: *P* < .001 and *P* < .001, respectively). This means that the significant correlation between cognition and survival was not explained by the presence of a severe complication, but rather appeared to be caused by the direct effect of cognition on survival. All effect estimates showed positive correlations between total number of impaired domains and mortality. So, patients with multiple impaired domains had a shorter survival than patients with one impaired domain. We also found a shorter survival in LGG patients with impaired attention and executive functioning (total effect: *P* < .001; direct effect: *P* < .001) and in HGG patients with impaired memory function (total effect: *P* = .001; direct effect: *P* = .003) compared to patients without these impaired domains. For the domain language in LGG patients, only the total effect was significant (*P* = .029). So, the significant total effect of impaired language function on survival could not be explained by either the direct effect of this impaired domain on survival or the presence of a severe complication.

**Table 5:** Results of Mediation Analyses for “Complication”

Neurocognitive deficit per domain		LGG		HGG	
Complication		*est DWLS (95%-CI)*	*P-value*	*est DWLS (95%-CI)*	*P-value*
Total impaired domains	Direct (c)	0.244 (0.034–0.454)	.023*	0.759 (0.631–0.886)	<.001*
	Indirect (a * b)	0.018 (–0.033 to 0.068)	.497	0.015 (–0.032 to 0.062)	.530
	Total (c + (a * b)	0.262 (0.048–0.475)	.016*	0.774 (0.654–0.894)	<.001*
Psychomotor speed	Direct (c)	0.270 (–0.113 to 0.652)	.167	0.016 (–0.266 to 0.297)	.913
	Indirect (a * b)	0.023 (–0.037 to 0.083)	.457	0.054 (–0.028 to 0.137)	.197
	Total (c + (a * b)	0.292 (–0.084 to 0.668)	.127	0.070 (–0.205 to 0.345)	.618
Visuospatial functioning	Direct (c)	–0.254 (–0.749 to 0.241)	.315	–0.147 (–0.466 to 0.172)	.366
	Indirect (a * b)	0.115 (–0.120 to 0.351)	.338	0.048 (–0.031 to 0.128)	.235
	Total (c + (a * b)	–0.139 (–0.541 to 0.264)	.499	–0.099 (–0.409 to 0.212)	.534
Language	Direct (c)	0.350 (–0.089 to 0.790)	.118	0.115 (–0.240 to 0.471)	.525
	Indirect (a * b)	0.021 (–0.181 to 0.222)	.840	0.075 (–0.030 to 0.181)	.163
	Total (c + (a * b)	0.371 (0.039–0.703)	.029*	0.190 (–0.129 to 0.510)	.242
Memory	Direct (c)	0.240 (–0.208 to 0.688)	.294	0.384 (0.131–0.636)	.003*
	Indirect (a * b)	0.034 (–0.221 to 0.289)	.792	0.038 (–0.025 to 0.101)	.238
	Total (c + (a * b)	0.274 (–0.064 to 0.612)	.112	0.422 (0.182–0.661)	.001*
Attention and executive functioning	Direct (c)	0.566 (0.299–0.834)	<.001*	0.136 (–0.133 to 0.404)	.322
	Indirect (a * b)	0.017 (–0.034 to 0.068)	.523	0.044 (–0.032 to 0.120)	.255
	Total (c + (a * b)	0.583 (0.319–0.847)	<.001*	0.180 (–0.085 to 0.445)	.184

Predictor variable (neurocognitive domains) are shown on the left, divided into three effect estimates: direct (c), indirect (a * b) and total effect (c + (a * b)). See Figure 1 for further explanation of these effects. Included mediator is severe complication, defined as “Common Terminology Criteria for Adverse Events score of ≥3”. Analyses were performed in low-grade glioma (LGG) and high-grade glioma (HGG) subgroups separately. LGG corresponds to grade II/III astrocytoma IDH-mutated and grade II/II oligodendroglioma 1p19q deletion. HGG corresponds to grade II/III astrocytoma IDH-wildtype, glioblastoma IDH-mutated and IDH-wildtype. Estimates are reported as diagonally weighted least squares (est DWLS) with 95% confidence interval (CI) and *P*-value. Significant *P*-values (≤.05) are marked with an asterisk(*).

## Discussion

Several studies have shown that cognitive impairments are independently associated with shorter survival time in diffuse glioma patients,^23^ even after correction for contemporary (WHO 2016) histomolecular diagnosis.^3^ One possible explanation for this robust relationship is a practical one: cognitively impaired patients influence the choice and course of treatment. In this study, we investigated whether this relationship between cognition and survival was mediated by three different factors: undertreatment, complications, and compliance. We hypothesized that patients with cognitive impairment may undergo less intensive treatment, be less compliant, and suffer more from complications and that these factors could consequently lead to shortened survival times. As in previous studies, we investigated this in both HGG and LGG separately and used disease-specific thresholds in both study populations.^11^ Indeed, we confirmed the significant association between different cognitive domains and survival. However, none of these associations could be explained by one of the aforementioned intermediate factors. This suggests that other mechanisms should be involved in the relation between cognition and survival.

In their paper on the relationship between cognitive functioning and survival of glioma patients, Johnson & Wefel suggested that physicians, consciously or unconsciously, recommend patients with poor cognitive functioning less aggressive treatment at the time of diagnosis than patients with good cognitive functioning.^24^ However, this suggestion was not based on empirical evidence and the nature and consequences of this relation were not investigated further in their study. To our knowledge, no data specific for diffuse glioma has been published on this topic previously. We found that HGG patients with impaired cognitive functioning had an increased risk of undertreatment, as well as an increased risk of non-compliance. In other chronic diseases as hypercholesterolemia, multiple studies showed a comparable relationship between cognition on the one hand, and undertreatment and adherence to medication on the other.^25^

To our knowledge, no glioma-specific research about higher risk of complications in cognitively impaired patients has been published yet. We found that HGG patients with impairments in the domain language had an increased risk of severe complications during follow-up. However, after adjusting for confounders, there was no significant association between cognition (any domain) and the occurrence of severe complications. Since we performed the mediation analysis with inclusion of the same confounders, this could be the reason there were no significant effects of this intermediate factor in the mediation analyses. In other words, the association we found between cognition and complications is a mixed effect, which was biased by confounders. Overall, complications did not mediate the relationship between cognition and survival in our analysis.

Apparently, the abovementioned mediators *cannot* sufficiently explain the relation between cognition and survival. So, the question arises: what mechanisms *do* explain this association? In an earlier study we discussed the notion of cognitive functioning as a marker for diffuse tumor infiltration, and the option that cognitive functioning and survival are determined by overlapping germline and somatic molecular-genetic factors.^3^ Indeed, several studies already showed that multiple SNPs (single nucleotide polymorphisms) are associated with neurocognitive performance^26,27^ and cognitive impairment is found to occur more frequently in glioma patients with wild-type *IDH1* tumor status when compared to those with a mutant *IDH1* tumor status.^28,29^ A more recent publication of Daniel et. al. showed that intratumoral connectivity strength in functionally intact regions may persist within glioblastoma and is a prognostic marker for overall survival.^30^ Two regions are considered to show functional connectivity if there is a statistical relationship between the measures of activity recorded for them. In this study it was measured within tumor boundaries with resting-state functional MRI (rs-fMRI) and relies on identification of temporally correlated, intrinsic fluctuations of infra-slow blood-oxygen-level-dependent (BOLD) signals (ie, functional connectivity [FC]). Hypothetically, functional intratumoral connectivity can act as a confounder or intermediate factor in the relationship between cognition and survival. Network disruption by the tumor on a global, whole-brain level (so beyond the tumor boundaries) is related to survival and is associated with cognitive functioning as well. Having lower *functional (alpha) connectivity* relates to poorer cognitive performance in patients with diffuse glioma, regardless of age, education, and presence of epilepsy.^31^ Increases in alpha band connectivity corresponds to improved cognitive functioning.^32^ Possibly, *global connectivity* is related to survival as well and can as such interact in the relation between cognition and survival. Or, the link between brain signaling and survival may be found in recent landmark studies, showing that neurons and glioma cells have direct synaptic interactions, and that increased neuronal signaling stimulates glioma proliferation.^33–35^

A cardinal feature of our mediation analysis concerns the inclusion of relevant confounders. The choice of confounders is based on the current literature, both for LGG and HGG, and previous studies conducted in our study population.^3^ Moreover, we only adjusted for true confounders, i.e. factors with a relationship with both the determinant (cognition) and the outcome (survival), since we wanted to evaluate the independent relation between NCF and survival. For this reason, several established prognostic/predictive factors were not included in our analyses: tumor location, steroid use, and extent of resection. The location of the tumor does not act as a confounder in the relationship between NCF and survival, since location itself (besides eloquent areas) does not predict survival in patients with diffuse glioma. Steroid use affects only indirectly NCF and survival via KPS and tumor volume, which we both included as confounders in our analyses. The same applies to extent of resection because the extent of resection could possibly influence patients’ survival but cannot affect preoperative cognitive functioning. We only adjusted for epilepsy at presentation in HGG patients, since the positive correlation between epilepsy and survival has only been demonstrated in glioblastoma patients.^14^

Limitations of our study should be mentioned as well. Cognitive functioning was routinely evaluated in patients undergoing awake surgery, leading to a selected subset of all glioma patients. Previous studies of our cohort show these patients may have different characteristics (e.g. tumor localization and clinical performance score) compared to patients undergoing non-awake surgery.^2,3^ On the other hand, all consecutive patients that underwent awake surgery were included regardless their preoperative cognitive performance. Therefore, our analyses should offer valid results, without selection bias or compromised internal validity of the study. Still, the patient selection could possibly affect the external validity of our study. However, baseline characteristics show a heterogeneous study population, with still a considerable percentage of patient with a poor prognosis (defined as grade II or III astrocytoma IDH-WT and GBM IDH-WT/M) and tumor location in the right hemisphere. Additionally, patients with KPS<60 are not suitable for any postoperative treatment, so in this patient group it is not possible at all to investigate “undertreatment”. For this reason, we think our results can be carefully extrapolated to non-awake operated patients too. Another limitation is the lack of a “pure” variable for the mediator compliance. The percentage of received chemotherapy and dose reduction are surrogate measures of compliance, but not direct measures of *patient’s* compliance per se. Dose reduction is often a doctors’ choice, for example in case of a complication, although the physician may decide on the severity and consequences of side effects during shared decision making with the patient. In addition, the percentage of received chemotherapy might be less than 100% if a complication occurs and therefore therapy is switched or stopped. However, our results show no indirect significant effects for either compliance or complications as intermediate factor, so the possible overlap in variables used as mediator variables is less relevant. Regarding the variable undertreatment, other factors that are not included in formal criteria, e.g. physical co-morbidities, could also have influenced the choice of therapy, in addition to cognitive impairments. Thus, the variable undertreatment does not directly reflect undertreatment solely caused by cognitive impairment. It is unlikely, though, that these other factors affected the path between cognitive impairments and undertreatment. The exact mechanisms through which baseline cognitive dysfunction may influence treatment choice and course are still unknown and may be complex. Of note, one possible contributing factor may be that patients with baseline cognitive dysfunction may be more vulnerable to additional cognitive deterioration during treatment, further complicating their capability to undergo treatment, e.g. at recurrence. We limited our analysis to baseline cognition, as this is the established risk factor for survival. Future prospective studies, e.g. with repeated neuropsychological testing and registration of reasons for clinical decisions during the treatment course, could offer further insight into how cognitive functioning may evolve and may influence aspects of clinical management.

Moreover, caution is warranted in extrapolating our results of a single-center study to other centers that may adopt different treatment guidelines, potentially biasing the variable undertreatment. Despite this single-center setting, the application of a consistent treatment protocol, and the systematic analysis we used, brings validity that should extend beyond the context of this institution.

In summary, we aimed to investigate whether therapeutic decision-making, compliance, and complications act as intermediate factors in the established relation between cognitive functioning and survival in patients with diffuse gliomas. We confirmed the hypothesis that patients with cognitive impairment may undergo less intensive treatment, be less compliant, and suffer from complications more frequently. However, these three factors do not contribute in the relation between cognition and survival. Further research should investigate other explanatory mechanisms for this relationship, including overlap in causative molecular-genetic factors and pathways, as well as intratumoral and global connectivity.

## Supplementary Material

npac027_suppl_Supplementary_Figure_S1

npac027_suppl_Supplementary_Figure_S2

npac027_suppl_Supplementary_Material
