# Supplementary figures and images for: Complications, compliance, and undertreatment do not explain the relationship between cognition and survival in diffuse glioma patients
Source: Neurooncol Pract. 2022 Apr 5;9(4):284–98. doi: 10.1093/nop/npac027 (PMC9290897; doi:10.1093/nop/npac027)

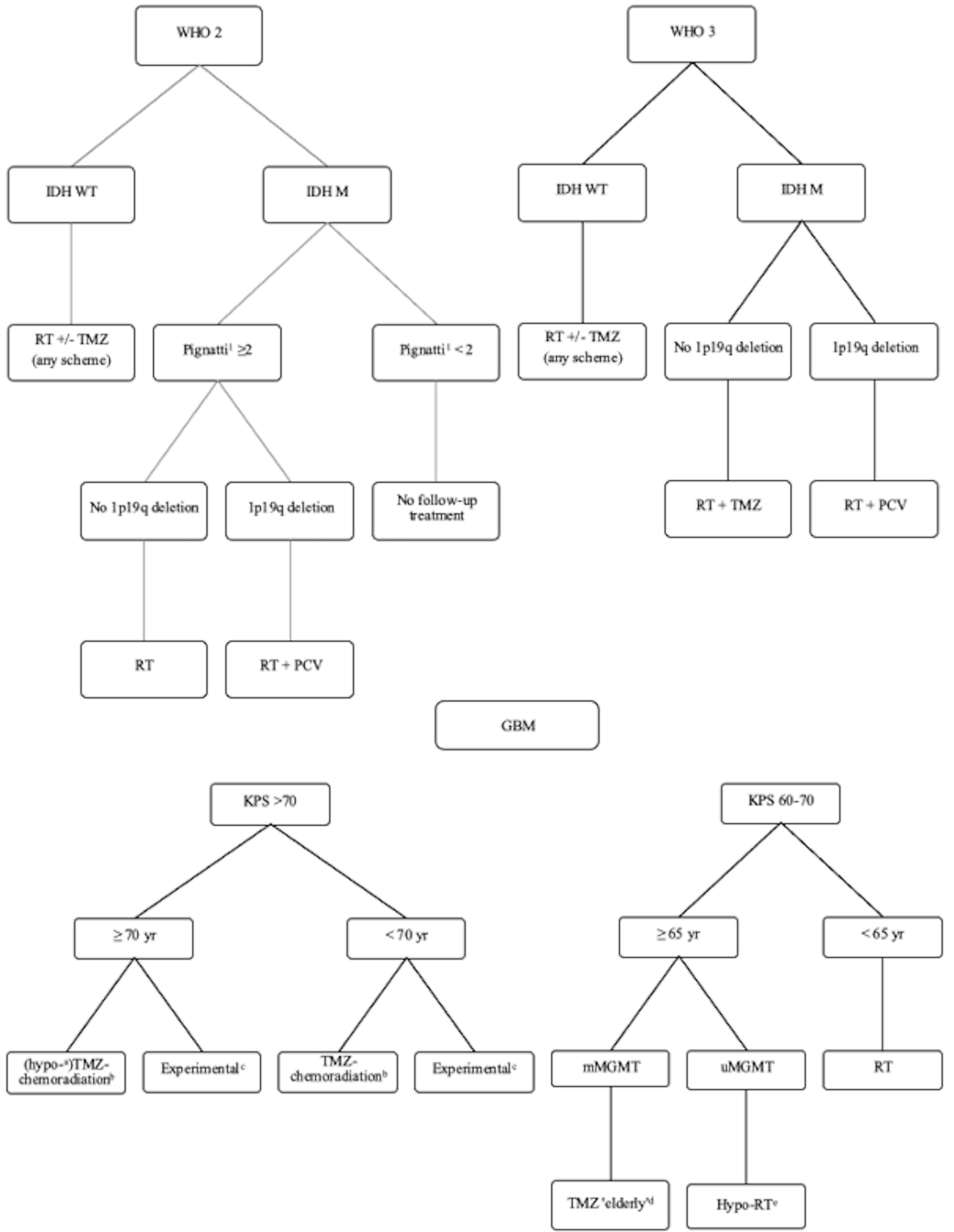

Supplement: npac027_suppl_Supplementary_Figure_S1 [file npac027_suppl_supplementary_figure_s1.jpeg]

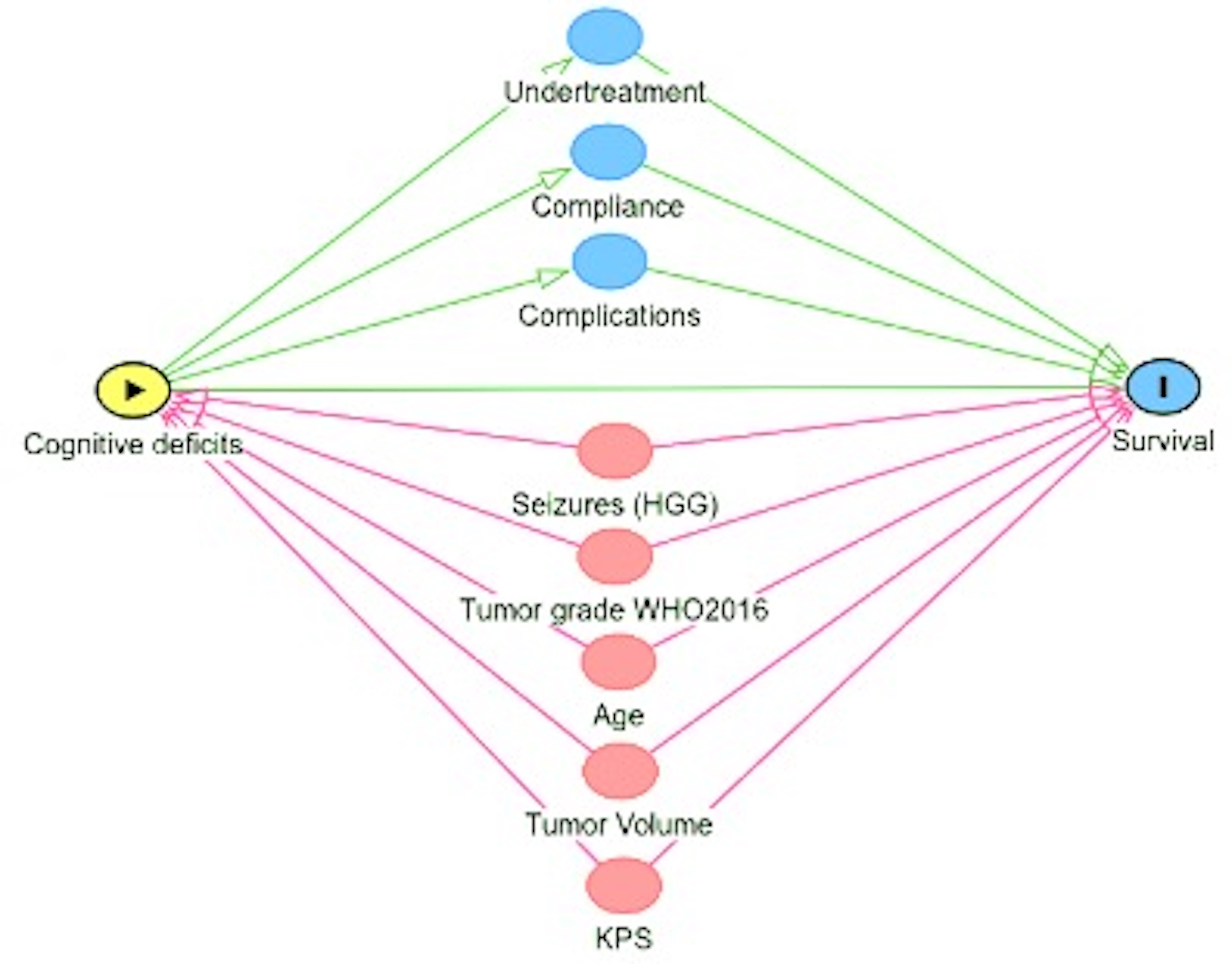

Supplement: npac027_suppl_Supplementary_Figure_S2 [file npac027_suppl_supplementary_figure_s2.jpeg]
