# Supplementary material for: Complications, compliance, and undertreatment do not explain the relationship between cognition and survival in diffuse glioma patients
Source: Neurooncol Pract. 2022 Apr 5;9(4):284–98. doi: 10.1093/nop/npac027 (PMC9290897; doi:10.1093/nop/npac027)
Supplement: npac027_suppl_Supplementary_Material [file npac027_suppl_supplementary_material.docx]

**Supplementary material**

Methods Supplement: neuropsychological tests

Supplementary Table 1: Neuropsychological tasks per domain

Supplementary Table 2: Covariates included in the mediation model, per glioma subgroup

Supplementary Table 3: Complication main groups

Supplementary figure 1: Flowcharts for follow-up treatment for all different glioma grades(II-IV), as used in the University Medical Centre Utrecht

WHO = World Health Organization; IDH = Isocitrate dehydrogenase; RT = Radiotherapy; TMZ = Temozolomide chemotherapy; PCV = Procarbazine, Lomustine, Vincristine chemotherapy; WT = Wildtype; M = Mutant; GBM = glioblastoma; KPS = Karnofsky Performance Scale; mMGMT = methylated O6-methylguanine-DNA methyltransferase; uMGMT = unmethylated O6-methylguanine-DNA methyltransferase; TMZ ‘elderly’ = Temozolomide chemotherapy elderly scheme; Hypo-RT = Hypo-fractionated radiation; BSC = Best Supportive Care;

^a^Hypo-TMZ-chemoradiation: RT, concurrent TMZ daily, adjuvant TMZ

^b^TMZ-chemoradiation: RT, concurrent TMZ daily, adjuvant TMZ

^c^Experimental: any form of clinical experimental study for diffuse glioma conducted within the UMCU

^d^TMZ ‘elderly’: 6 cycles TMZ according to Wick W et al. Lancet Oncol 2012; 13: 707-715

^e^Hypo-RT: hypofractionated radiotherapy

1. Pignatti F, van den Bent M, Curran D, et al. Prognostic factors for survival in adult patients with cerebral low-grade glioma. *J Clin Oncol.* 2002; 20(8):2076-2084.

Supplementary figure 2: Directed acyclic graph of all the possible relations between covariates, determinant and outcome

HGG = high grade glioma; KPS = Karnofsky Performance Scale.

**Methods Supplement: neuropsychological tests**

Neuropsychological tests often tap into more than one cognitive domain and classification into cognitive domains often varies in the literature. We made use of a predetermined test classification in accordance with previous studies from our group and literature (Supplementary Table 1)^1-3^. The neuropsychological evaluation was conducted by an experienced neuropsychologist shortly (1-7 days) before the awake brain tumor surgery. Each neuropsychological test was scored according to standardized scoring criteria. For normative comparisons the unadjusted scores were transformed into Z-scores based on the mean and standard deviation of control subjects derived from published, age- and education-specific, norm data.

We measured NCF data at the individual patient-level, which means that we counted the number of individual patients with an impaired performance per domain. For high-grade glioma (HGG), a patient was considered impaired for a given domain if the patient performed below -2 SD on any of the administered (sub)tests within that domain, in accordance with previous studies and based on clinical practice^4^. Because of the lower frequencies of impairments in low-grade glioma (LGG) patients, we used a threshold of -1.5 SD for cognitive deficits in each domain. This slightly more liberal threshold was chosen, in accordance with previous studies from our group and other literature, to optimize clinical relevance for LGG patients. In general, LGG patients are cognitively less affected than HGG patients. Though, since they are generally young (20-45 years at debut) and socially and professionally active, relatively mild cognitive symptoms may have severe impact on daily life^5, 6^.

**Supplementary Table 1: Neuropsychological tasks per domain**

| Attention & Executive Functioning |
| --- |
| Wechsler Adult Intelligence Scale (WAIS) Digit Span Forward^a^  Trail Making Test (TMT) Switching ratio (TMTB/TMTA)^b^  Phonologic Fluency^c^  Stroop/Delis Kaplan Executive Function System (DKEFS) inhibition ratio^d^  Wechsler Adult Intelligence Scale (WAIS) Digit SpanBackward |
| Memory |
| RAVLT-Dutch Version immediate, delay, recognition^e^  Rey-Osterieth Complex Figure Test (ROCF) delay^f^  Semantic Fluency^g^ |
| Visuospatial functioning |
| Judgment of Line Orientation (JOLO)^h^  ROCF direct |
| Psychomotor Speed |
| Stroop/DKEFS I  Stroop/DKEFS II  TMTA |
| Language |
| Boston Naming Test^i^  Token Test^j^ |

1. Wechsler Adult Intelligence Scale Third Edition Digit Span [WAIS-III] (WAIS-III Administration and scoring manual, 1997), Wechsler Adult Intelligence Scale Fourth Edition Digit Span [WAIS-IV] (WAIS-IV-NL Technische handleiding, 2013)
2. Trail Making Test [TMT] (Giovagnoli, Del Pesce, Mascheroni, Simoncelli, Laiacona, & Capitani, 1996)
3. Phonologic Verbal Fluency Test [Lexical Fluency] (Harrison, Buxton, Husain, & Wise, 2010; Schmand, Groenink, & Van Den Dungen, 2008)
4. Delis-Kaplan Executive Function System [DKEFS] (Delis, D. C., Kaplan, E., & Kramer, J. (2001)
5. 15 Words Test [15WT] (Saan & Deelman, 1986)
6. Rey-Osterieth Complex Figure Test [ROCF] (Berry & Carpenter, 1992; Spreen & Strauss, 1998)
7. Semantic Verbal Fluency Test [Semantic Fluency] (Harrison et al., 2010). Semantic Fluency was classified as a measure of memory based on Kavé et al., 2020 and Henry et al., 2004. ^7, 8^
8. Judgment of Line Orientation [JULO] (Benton, Sivan, Hamsher, Varney, & Spreen, 1994; Benton, Varney, & Hamsher, 1978)
9. Boston Naming Task [BNT] (Heesbeen & Van Loon-Vervoorn, 2001)
10. Token Test [TT] (Boller & Vignolo, 1966)

**Supplementary Table 2: Covariates included in the mediation model, per glioma subgroup.**

| **Low grade glioma** | **High grade glioma** |
| --- | --- |
| Age at awake surgery  Pre-operative tumor volume  Karnofsky performance scale  WHO 2016 classification | Age at awake surgery  Pre-operative tumor volume  Karnofsky performance scale  WHO 2016 classification  Epilepsy at presentation |

Low grade glioma are Grade II/III Astrocytoma IDH-mutated and Grade II/II Oligodendroglioma 1p19q deletion. High grade glioma include Grade II/III Astrocytoma IDH-Wildtype and Glioblastoma IDH-mutated and IDH-Wildtype.

**Supplementary table 3: Complication main groups**

| **Main group** | **Complication** |
| --- | --- |
| *General* | Pain Fatigue  Alopecia  Wound leakage |
| *Infectious* | Urine tract infection  Pneumonia  Fever  Infection of other cause |
| *Coagulation* | Deep vein thrombosis  Pulmonary embolism |
| *Neurological (central)* | Cerebral infarction  Cerebral bleeding  Cerebral edema  Cyst  Cognitive decline  Iatrogenic  Seizure |
| *Neurological (peripheral)* | Paresis e.c.i.*  Sensory impairment  Neuropathy |
| *Gastrointestinal* | Nausea / vomiting  Constipation |
| *Hematological* | Thrombocytopenia  Neutropenia |
| *Skin* | Allergic skin reaction  Itch |
| *Other causes* | All other causes |

*if cause of paresis is found, the cause is scored as the complication.

References

1. Nys GM, van Zandvoort MJ, de Kort PL, Jansen BP, de Haan EH, Kappelle LJ. Cognitive disorders in acute stroke: prevalence and clinical determinants. Cerebrovasc Dis 2007;23:408-416.

2. Biesbroek JM, van Zandvoort MJ, Kappelle LJ, Velthuis BK, Biessels GJ, Postma A. Shared and distinct anatomical correlates of semantic and phonemic fluency revealed by lesion-symptom mapping in patients with ischemic stroke. Brain Struct Funct 2016;221:2123-2134.

3. Doherty JM, Belletier C, Rhodes S, et al. Dual-task costs in working memory: An adversarial collaboration. J Exp Psychol Learn Mem Cogn 2018.

4. van Kessel E, Emons MAC, Wajer IH, et al. Tumor-related neurocognitive dysfunction in patients with diffuse glioma: a retrospective cohort study prior to antitumor treatment. Neuro-Oncology Practice 2019;6:463-472.

5. van Kessel E, Emons MAC, Wajer IH, et al. Tumor-related neurocognitive dysfunction in patients with diffuse glioma: a retrospective cohort study prior to antitumor treatment. Neuro-Oncology Practice 2019.

6. van Kessel E, Baumfalk AE, van Zandvoort MJE, Robe PA, Snijders TJ. Tumor-related neurocognitive dysfunction in patients with diffuse glioma: a systematic review of neurocognitive functioning prior to anti-tumor treatment. J Neurooncol 2017;134:9-18.

7. Kave G, Sapir-Yogev S. Associations between memory and verbal fluency tasks. J Commun Disord 2020;83:105968.

8. Henry JD, Crawford JR, Phillips LH. Verbal fluency performance in dementia of the Alzheimer's type: a meta-analysis. Neuropsychologia 2004;42:1212-1222.
